# Long‐term intervention with transcranial magnetic stimulation in Primary Progressive Aphasia: a randomized, double‐blind, sham‐controlled clinical trial

**DOI:** 10.1002/alz70858_100326

**Published:** 2025-12-25

**Authors:** Lucia Fernandez‐Romero, María Nieves Cabrera‐Martín, Cristina Delgado‐Alonso, Paz Suárez‐Coalla, Stephanie M Grasso, Antonio Portolés, Natalia Pérez‐Macías, María Teresa Carreras, María Díez‐Cirarda, María José Gil‐Moreno, Javier Olazarán, Alba Vieira, Silvia Oliver‐Mas, Ulises Gómez‐Pinedo, Jorge Matias‐Guiu, Jordi A Matias‐Guiu

**Affiliations:** ^1^ Hospital Clinico San Carlos, Madrid, Madrid, Spain; ^2^ Hospital Clinico San Carlos, Madrid, Spain; ^3^ Universidad de Oviedo, Oviedo, Spain; ^4^ The University of Texas at Austin, Austin, TX, USA; ^5^ Hospital Universitario La Princesa, Madrid, Spain; ^6^ Hospital Clínico San Carlos, Madrid, Madrid, Spain; ^7^ Hospital General Universitario Gregorio Marañón, Madrid, Spain; ^8^ Hospital Clinico Universitario San Carlos, Madrid, Madrid, Spain

## Abstract

**Background:**

Primary progressive aphasia (PPA) is a neurodegenerative syndrome characterized by language impairment there is currently no effective treatment. Non‐invasive techniques like transcranial magnetic stimulation (TMS) have shown potential in improving symptoms, but their long‐term effects in PPA are unknown. This study aimed to evaluate the efficacy of TMS combined with language therapy in PPA at 6 months.

**Method:**

This double‐blinded, prospective, randomized, parallel clinical trial enrolled 63 PPA patients (24 nonfluent PPA (nfvPPA), 12 semantic PPA (svPPA) and 27 logopenic (lvPPA)). Participants were randomized in a 2:1 ratio to either active TMS or sham TMS. The intervention involved a 6‐month intermittent theta‐burst stimulation protocol over the left dorsolateral prefrontal cortex (DLPFC) followed by language therapy based on Lexical Retrieval Cascade Treatment. The primary outcome was the standardized uptake value (SUV) in a brain region associated with PPA progression, measured at baseline and at 6 months using FDG‐PET. Secondary outcomes included language performance (Mini‐Linguistic State Examination, word naming, words per minute in spontaneous speech), functional independence, and neuropsychiatric symptoms, assessed at baseline, 3 months, and 6 months.

**Result:**

The primary outcome showed a significant benefit in the active TMS group compared to the sham group at 6 months. Active TMS also improved secondary outcomes, including language abilities, functional independence, and neuropsychiatric symptoms, except in words per minute during spontaneous speech. Adherence was complete in 92% of participants.

**Conclusion:**

Intermittent theta‐burst stimulation combined with language therapy for 6 months was associated with reduced decline in regional brain metabolism and improvements in language performance, functional independence, and neuropsychiatric symptoms in patients with PPA.

